# Predicting mechanism of action of novel compounds using compound structure and transcriptomic signature coembedding

**DOI:** 10.1093/bioinformatics/btab275

**Published:** 2021-07-12

**Authors:** Gwanghoon Jang, Sungjoon Park, Sanghoon Lee, Sunkyu Kim, Sejeong Park, Jaewoo Kang

**Affiliations:** Department of Computer Science and Engineering, Korea University, Seoul, Republic of Korea; Department of Computer Science and Engineering, Korea University, Seoul, Republic of Korea; Department of Computer Science and Engineering, Korea University, Seoul, Republic of Korea; Department of Computer Science and Engineering, Korea University, Seoul, Republic of Korea; Department of Computer Science and Engineering, Korea University, Seoul, Republic of Korea; Department of Computer Science and Engineering, Korea University, Seoul, Republic of Korea; Interdisciplinary Graduate Program in Bioinformatics, Korea University, Seoul, Republic of Korea

## Abstract

**Motivation:**

Identifying mechanism of actions (MoA) of novel compounds is crucial in drug discovery. Careful understanding of MoA can avoid potential side effects of drug candidates. Efforts have been made to identify MoA using the transcriptomic signatures induced by compounds. However, these approaches fail to reveal MoAs in the absence of actual compound signatures.

**Results:**

We present MoAble, which predicts MoAs without requiring compound signatures. We train a deep learning-based coembedding model to map compound signatures and compound structure into the same embedding space. The model generates low-dimensional compound signature representation from the compound structures. To predict MoAs, pathway enrichment analysis is performed based on the connectivity between embedding vectors of compounds and those of genetic perturbation. Results show that MoAble is comparable to the methods that use actual compound signatures. We demonstrate that MoAble can be used to reveal MoAs of novel compounds without measuring compound signatures with the same prediction accuracy as that with measuring them.

**Availability and implementation:**

MoAble is available at https://github.com/dmis-lab/moable

**Supplementary information:**

Supplementary data are available at *Bioinformatics* online.

## 1 Introduction

Elucidating mechanism of actions (MoA) of compounds is important in drug discovery process. Predicting accurate MoAs of compounds can not only improve on-target efficacy but also avoid potential side effects caused by off-target effects. Without the proper understanding of MoAs, the success rate of clinical trials of drug candidates can decrease (Bantscheff *et al.*, 2007; Editorial, 2010; Wehling, 2009).

One approach to identify MoA of compounds is to conduct binding affinity assays. This approach can help to identify target proteins that physically bind to compounds (Davis *et al.*, 2011). However, experimental assays are usually expensive and time-consuming. As an alternative, computational approaches such as molecular docking or machine learning methods can be used (Li *et al.*, 2020; Öztürk *et al.*, 2018; Trott and Olson, 2010). However, MoAs based on binding affinity only focus on the physical interaction between compounds and target proteins, which neglects the impact of compounds on cellular phenotype (e.g. transcriptomic signatures).

To date, Connectivity Map (CMap) and Library of Integrated Network-based Cellular Signatures (LINCS) have provided large-scale transcriptomic signature datasets, where the transcriptomic signatures represent differential gene expression profiles in response to cellular perturbation (Lamb *et al.* 2006; Subramanian *et al.*, 2017). The LINCS/L1000 dataset includes 473 647 transcriptomic signatures corresponding to 25 200 perturbagens (e.g. chemical compounds, gene knock-down) (Subramanian *et al.*, 2017). This large-scale transcriptomic signature dataset has provided opportunities to identify MoAs of compounds reflecting the transcriptomic cellular phenotype (Keenan *et al.*, 2019; Musa *et al.*, 2018; Pilarczyk *et al.*, 2019). To reveal MoAs using transcriptomic signatures, one major approach is to identify pathways whose activities are significantly affected by compound treatment. Gene set enrichment analysis (GSEA) can be applied to infer pathway activity scores by computing enrichment scores. The enrichment score indicates over-representation of pathway genes in highly differentially expressed genes (Khatri *et al.*, 2012; Reimand *et al.*, 2019; Subramanian *et al.*, 2005).

Recently, several works (Dugourd and Saez-Rodriguez, 2019; Ren *et al.*, 2020; Schubert *et al.*, 2018) have claimed the limitation of computing pathway activity scores using the transcriptomic signatures of pathway genes. This is because gene expression is the consequence of pathway activity and an altered pathway activity is rarely represented in transcriptomic signatures of pathway genes. Rather, the pathway activity is usually determined by posttranslation modification (e.g. phosphorylation, acetylation) of signaling proteins constituting the pathway. Thus, mapping transcriptomic signatures of pathway genes to compute pathway activities can produce misleading target pathway hypotheses (Szalai and Saez-Rodriguez, 2020).

To overcome the challenge, several efforts have been made to utilize the transcriptomic signatures of genetic perturbation (GP) such as gene knock-down and gene knock-out to infer pathway activity (Pilarczyk *et al.*, 2019; Ren *et al.*, 2020). In the iLINCS study (Pilarczyk *et al.*, 2019), MoA pathways were identified based on the connectivity between compound signatures (i.e. transcriptomic signatures of a compound) and GP signatures (i.e. transcriptomic signatures of GP). Genes whose GP signatures are similar to a given compound signature were selected. Frequently selected genes can be viewed as potential targets of compounds. To predict MoAs, pathway enrichment analysis was performed based on the connected GP genes. Another recent work from Ren *et al.* (2020) also showed the effectiveness of using GP signatures in identifying pathway-level MoAs of compounds. The method computes pathway activity scores by combining the GP signatures of pathway genes. Both studies demonstrated that the connected GP signatures are more useful to identify pathway-level MoA of compounds than compound signatures.

Although the aforementioned studies opened a new avenue for predicting MoAs, these methods have a common limitation. They fail to predict MoAs when the compound signatures are absent. To address this limitation, we propose a novel method named MoAble that can identify MoAs of compounds without requiring actual compound signatures. We applied a coembedding technique to jointly encode chemical structures of compounds (referred to as compound structure) and compound signatures into the same latent embedding space. The coembedding enables generating embedding vectors from a compound structure where the embedding vector contains compressed representation of the compound signature. To predict pathway-level MoA using the embedding vectors, we perform pathway enrichment analysis based on the genes whose GP signature embeddings are highly connected with the compound structure embedding, where the GP signature embedding and compound structure embedding indicate the embedding vector of the GP signature and compound structure, respectively. Pathways that are over-represented with highly connected GP genes are interpreted as MoAs of compounds. First, using MoAble, we assess whether the coembedding model can generate reliable embedding vectors that can be used to predict MoAs. We quantitatively evaluate the prediction performance of MoAble by comparing with baseline methods that use actual compound signatures. We demonstrate that MoAble can achieve a similar prediction performance without using actual compound signatures as the baseline methods.

## 2 Materials and methods

**Fig. 1.**
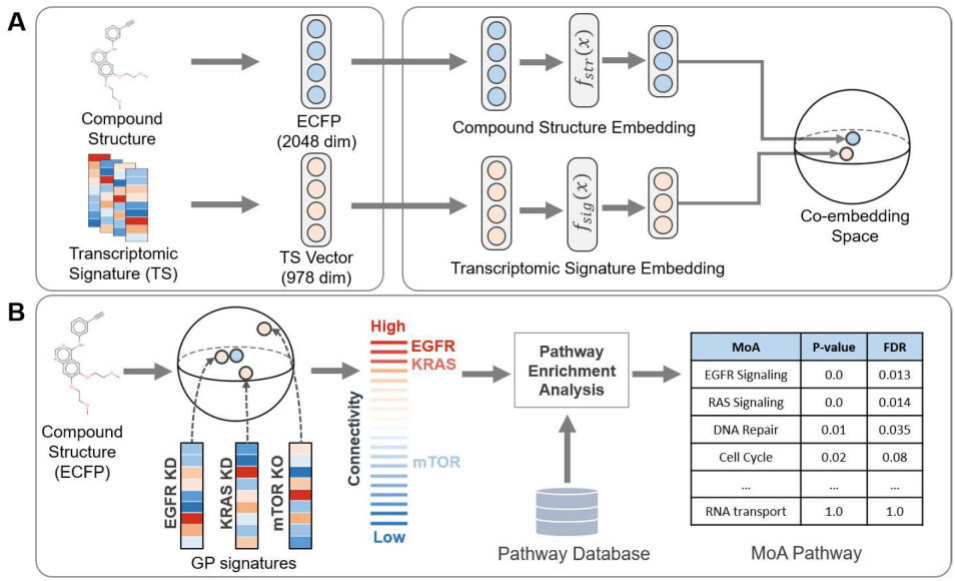
Overview of MoAble. (**A**) Coembedding model. The coembedding model takes a compound structure (as ECFP representation) and a compound signature as input. The coembedding model uses deep metric learning to jointly encode compound structures and signatures into the same embedding space. *f_str_* and *f_sig_* indicate the neural network encoders of a compound structure and a signature, respectively. (**B**) MoA prediction. MoAs of a given compound are identified based on the connectivity between compound structure embedding and GP signature embedding. GP signatures are mapped into the coembedding space using the signature encoder (*f_sig_*). KD and KO denote knock-down and knock-out, respectively. Pathway enrichment analysis is performed on the connected genes, and highly enriched pathways are considered to be MoAs of the compound

MoAble aims to predict the MoAs for novel compounds when the actual compound signatures are not available. Figure 1 illustrates the overview of MoAble. We trained a deep metric learning-based coembedding model that jointly encodes the compound structures and signatures induced by the compounds into the same embedding space (Fig. 1A). Subsequently, the coembedding model takes a new compound structure as an input and generates an embedding vector that represents the compressed representation of the signature induced by the compound. MoAble then predicts MoAs of the compound based on the connectivity between the compound and GP signatures in the embedding space (Fig. 1B).

### 2.1 Dataset

We obtained signatures of compounds and GP from the L1000 dataset provided by the LINCS project (Subramanian *et al.*, 2017). The L1000 dataset provides gene expression profiles at various normalization levels (levels 1–5). We used level 5 data that represents transcriptomic signatures for 978 landmark genes (i.e. differential gene expression in response to perturbation). Here, we denote transcriptomic signature as signature for brevity. We selected 282 038 compound signatures corresponding to 20 902 compounds and 192 068 GP signatures corresponding to 8741 genes. The types of GP include gene knock-down, gene knock-out and over-expression. Compound signatures were downloaded from Gene Expression Omnibus (accession number: GEO70138 and GEO92742) (Edgar *et al.*, 2002). GP signatures were downloaded from https://clue.io/data/CMap2020\#LINCS2020. For the chemical structure representation of compounds, we used 2048-bit extended-connectivity fingerprints (ECFP), which represents topological fingerprints of chemical compounds. Each bit in ECFP indicates the presence of a chemical substructure. We obtained the simplified molecular-input line-entry system (SMILES) representation of compounds provided in the L1000 dataset. We used RDKit (http://www.rdkit.org), an open-source cheminformatics software, to convert the SMILES representation to ECFP. For evaluation, we split the compound signatures as follows: 70% for training, 15% for validation and 15% for testing, with no overlapping compounds in each split dataset. We summarize the statistics of the dataset in Table 1.

**Table 1.** Statistics of compounds and GP signatures used in this work

	Compounds	Signatures
Compounds		
Training	14 631	201 286
Validation	3135	41 290
Test	3136	39 462
Total	20 902	282 038
	Target genes	Signatures
GP		
Gene knock-down	4345	36 720
Gene knock-out	5157	121 177
Over-expression	4040	34 171
Total	8741	192 068

### 2.2 Coembedding of compound structure and signature

Jeon *et al.* (2019) and Finlayson *et al.* (2020) have shown the effectiveness of deep metric learning to learn similarities between compounds (and between compounds and transcriptomic signatures) in the embedding space. Inspired by these works, we trained a deep metric learning-based coembedding model that can map compound structures and signatures induced by the compound into the same embedding space. Our method exploits the similarities between embedding vectors of GPs and those of compounds, so we choose a deep metric learning-based model which is specialized to measure the similarity relationship among embedding vectors. After training, it can generate embedding vectors that represent compressed signature information induced by the compound. Figure 1A illustrates an overview of the coembedding model. The coembedding model consists of two neural network encoders. The first encoder network, *f_str_*, maps input compound structure (ECFP) $X_{\mathrm{str}}\in R^{2048}$ to latent embedding vector $Z_{\mathrm{str}}\in R^{256}$. The second encoder network, *f_sig_*, maps input signature $X_{\mathrm{sig}}\in R^{978}$ to latent embedding vector $Z_{\mathrm{sig}}\in R^{256}$. Each encoder consists of four multilayer perceptron (MLP) layers with a rectified linear unit activation layers between the MLP layers. We set the number of hidden layer units for *f_str_* and *f_sig_* to 2048–512–256–256 and 512–512–256–256, respectively. To effectively encode compound structures and signatures simultaneously, encoders are optimized with triplet loss (Schroff *et al.*, 2015). To optimize embedding vectors with triple loss, three class of embedding vectors are required: *anchor*, *positive* and *negative*. Here, the compound signature embedding (*Z_s_*) is set to *anchor*, the compound structure embedding corresponding to the compound of *anchor* ($Z_{c}^{p}$) is set to *positive* and the compound structure embedding of any other compound ($Z_{c}^{n}$) is set to *negative*. Subsequently, we optimized the encoders as follows:

(1) $$\underset{\phi ,\psi }{\max}\sum_{i=1}^{N}\min(\text{sim}(Z_{sig_{i}},Z_{str_{i}^{p}})-\text{sim}(Z_{sig_{i}},Z_{str_{i}^{n}})-\alpha ,0)$$

where ϕ and *ψ* are the parameters of the two encoders and *α* denotes a margin indicating distance between *positive* and *negative* pairs. We define the distance between two embeddings as cosine similarity, and it is given as follows:

(2) $$\text{sim}(u,v)=\frac{u\cdot v}{\left||u||||v|\right|}$$.

The optimization procedure allows embedding vectors to be located close to each other in the embedding space if their signatures are similar. Also, the compound structures and signatures can be mapped into the same embedding space. This enables the generation of embedding vectors that contain signature information induced by the compound using its structure information alone.

We trained the coembedding model with the Adam optimizer (Kingma and Ba, 2014) using a learning rate of 0.0001. The minibatch size was set to 512. The hyperparameters were optimized using the validation compounds.

### 2.3 MoA identification

Figure 1B illustrates the MoA prediction procedure in MoAble. First, GP signatures are mapped to the coembedding space. Then, the connectivity between a compound embedding and GP embeddings are computed. Genes whose GP embeddings are highly connected with compound embedding can be viewed as potential targets for the corresponding compound. For each compound, pathway enrichment analysis is performed based on the connectivity scores of GP genes. Highly enriched pathways are considered as potential MoAs of the compound.

#### 2.3.1 Generating GP embedding

After training the coembedding model, we generate embedding vectors of GP signatures. GP signatures indicate signatures after perturbation of gene knock-down, gene knock-out and over-expression experiments. The GP signature is a 978-dimensional vector $X_{g}\in R^{978}$, that has the same dimension as the compound signature. We used signature encoder *f_s_* to generate the GP signature embedding vectors $Z_{g}\in R^{256}$.

#### 2.3.2 Connecting compound and GP

We computed the connectivity score between a given compound structure and GP signatures using their embedding vectors. Herein, we define connectivity as the cosine similarity between a compound structure embedding and a GP embedding vector. The range of cosine similarity is [−1, 1], where a higher score indicates that two embedding vectors represent similar signatures. There can be multiple GP signatures targeting the same gene. For example, in the L1000 dataset, knock-down experiments of mTOR were conducted in diverse cell lines. To connect a compound with GP signatures at a gene level, we only select the highest connected GP embedding for each gene. Connectivity score between compound *c* and GP gene *g* is defined as follows:

(3) $$S_{c,g}=\max{\left\{\text{sim}(Z_{c},Z_{g}^{i})\right\}}_{i=1}^{N}$$

where *N* is the number of GP signatures targeting the gene *g*. Finally, we could obtain the connectivity scores corresponding to 8741 genes for each compound.

#### 2.3.3 MoA pathway prediction

In this study, we define MoAs as pathways whose activity is highly affected by the compound treatment. For each compound, we rank GP genes based on the connectivity scores with the corresponding compound structure embedding. Pathway enrichment analysis was performed with the ranked GP genes. Highly enriched pathways with statistical significance [e.g. false discovery rate (FDR) < 0.1] are considered as MoAs of a given compound. We used a conventional GSEA method (Subramanian *et al.*, 2005) for pathway enrichment analysis and used KEGG_2019 as pathway gene set. However, various GSEA methods (Kuleshov *et al.*, 2016; Reimand *et al.*, 2016) and pathway gene sets (Fabregat *et al.*, 2018; Martens *et al.*, 2021) can be applied.

## 3 Results

### 3.1 Assessment of coembedding model for MoA identification

**Fig. 2.**
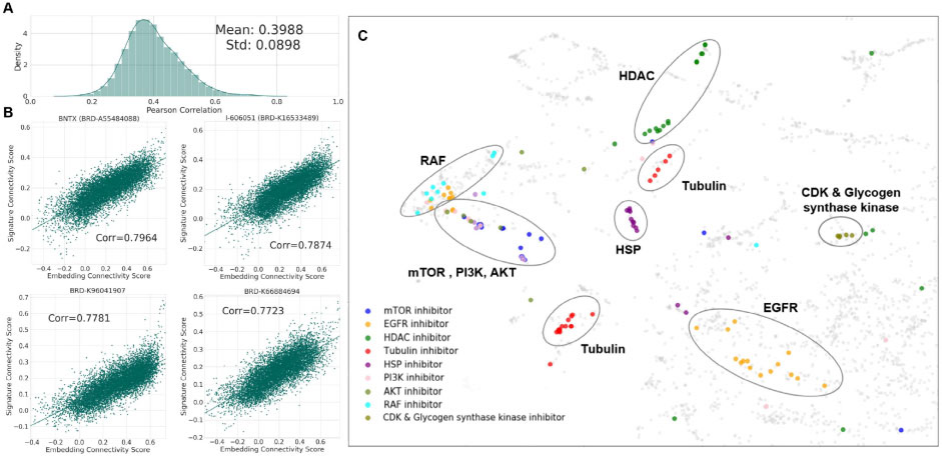
Assessment of coembedding model for MoA identification. (**A**) Distribution of correlation between connectivity scores of actual signatures and those of embeddings. The mean and std of correlations are 0.3988 and 0.0898, respectively. (**B**) Regression plots of four compounds with high correlation, BTNX, I-606051, BRD-K96041907 and BRD-K66884694. The *x* axis and *y* axis indicate the connectivity score of embeddings and that of actual signatures. (**C**) Two-dimensional representation of compound structure embedding vectors. The compounds targeting the same or similar MoA are circled

We assessed whether connectivity associations between compound signatures and GP signatures can be reproduced in the coembedding space ($R^{256}$). We calculated connectivity scores (i.e. cosine similarities) between compound signatures and GP signatures, which produced 8741 connectivity scores for each compound. We also measured connectivity scores between compound structure embeddings and GP signature embeddings. We calculated the correlations between the connectivity scores of actual signatures and the connectivity scores of embeddings. Figure 2A illustrates the correlation distributions across 20 902 compounds in L1000 (Pearson correlation, mean: 0.3988, std: 0.0898, *P*-value: 1.098e−30) which highlights the strong correlation between actual signature and embeddings. Our results show that connectivity associations between compounds and GP can be retained in the embedding vector space.

We also evaluated whether the coembedding model could generate embedding vectors that are informative for MoA identification. After training the coembedding model, we obtained compound structure embedding vectors of 20 902 compounds provided in L1000 dataset. UMAP (McInnes *et al.*, 2018) was used to visualize compound structure embeddings in a low-dimensional vector space (Fig. 2C). Each point indicates a two-dimensional representation of compound structure embedding. We investigated whether clusters observed in the UMAP plot share similar MoAs. We obtained MoA labels for 5552 compounds from clue.io (https://clue.io/). Examples of MoA labels are ‘AKT inhibitor’, ‘mTOR inhibitor’ and ‘EGFR inhibitor’. We can observe that compounds with similar MoAs are aligned close to each other. For example, mTOR inhibitors, PI3K inhibitors and AKT inhibitors are clustered in the embedding space. Interestingly, mTOR, AKT and PI3K are topologically related to each other in signaling pathways. Overall, our analysis shows the coembedding model’s ability to generate compound embedding vectors useful for MoA identification.

### 3.2 Evaluation of MoA prediction

#### 3.2.1 Baseline methods

MoAble uses compound structures and generates embedding vectors that contain compressed information of compound-induced signatures. It predicts MoAs based on the compound structure embedding. Thus, our proposed method does not require actual signature of compounds. We compare MoAble with methods that use actual signature when predicting MoAs. We use three baseline methods: TS-978, TS-12328 and TS-connectivity. TS-978 and TS-12328 directly use signature values to perform pathway enrichment analysis. TS-978 and TS-12328 use the signature values of 978 landmark genes and 12 328 genes, respectively. Note that, in the L1000 dataset, signatures corresponding to 978 landmark genes are only experimentally measured. From 978 genes, 12 328 signatures are mathematically inferred. TS-connectivity is similar to the iLINCS method (Pilarczyk *et al.*, 2019) where pathway enrichment analysis is performed based on connected GP signatures. We use cosine similarity to compute the connectivity between a compound and a GP signature. The difference between TS-connectivity and our method is that TS-connectivity uses actual signatures to compute connectivity scores between a compound and GP signatures whereas our method uses embedding vectors of a compound structure and GP signatures.

#### 3.2.2 Quantitative evaluation

In order to quantitatively evaluate the performance of MoA prediction, MoA labels of compounds were required. We obtained the MoA labels from KEGG Drug database (Kanehisa *et al.*, 2017). KEGG Drug database provides target pathways of compounds and we used the target pathways as true MoA labels. We could obtain true MoA labels for 4412 compounds. Of the 4412 compounds, 940 compounds were overlapped with L1000 compounds. As transcriptomic signatures for 940 compounds were available, we could use the 940 compounds to quantitatively compare our method with the baseline methods. We provide the list of compounds and MoA labels used in this study in Supplementary Data.

To quantitatively evaluate the performance of MoA prediction, we followed the evaluation criteria described in Nguyen *et al.* (2019). We evaluated the ability of MoA prediction models to identify true MoA pathways of compounds. Each MoA prediction model predicted MoA pathways with *P*-values, where pathways with low *P*-values are considered to be the candidates of MoAs.

**Fig. 3.**
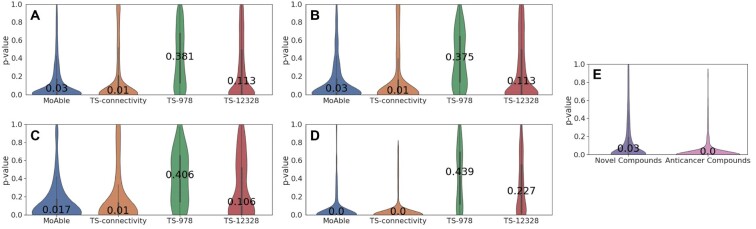
*P*-value distribution of true MoA pathways. For each method, resulting *P*-values of true MoA pathways are illustrated in violin plots. We compared MoAble with the three baseline methods: TS-connectivity, TS-978, TS-12328. (**A**) *P*-value distribution of true MoA pathways across 940 evaluation compounds. (**B**) *P*-value distribution across 283 unseen compounds. (**C**) *P*-value distribution across 28 unseen-hard compounds. (**D**) *P*-value distribution across 88 anticancer compounds. (**E**) *P*-value distribution across 2674 novel compounds and 94 novel anticancer compounds

Figure 3A shows violin plots indicating the distribution of *P*-values of true MoA pathways across 940 evaluation compounds. MoAble (median *P*-value = 0.03) achieved much lower median *P*-value than TS-978 (median *P*-value = 0.381), TS-12328 (median *P*-value = 0.113), and was comparable to TS-connectivity (median *P*-value = 0.01). We observed that the connectivity-based methods (MoAble, TS-connectivity) are more effective than methods using compound signature directly in terms of identifying MoA pathways. Although we did not use actual signatures to predict MoAs, we found MoAble to show predictive power similar to that of TS-connectivity that used actual signatures.

To evaluate MoAble’s performance on novel compound structures, we validated MoAble using 283 compounds that were previously excluded from the training set. We defined the 283 compounds as ‘unseen’ compounds. When we examined the structural similarities between training compounds and unseen compounds, most of the unseen compounds showed low similarities to training compounds (mean = 0.1418, SD = 0.0721, Supplementary Fig. S1). Tanimoto similarity was used to measure the structural similarity. We further defined ‘unseen-hard’ as the compound set in the unseen compounds with the similarity <0.3 to training compounds. It was highly selective to obtain the ‘unseen-hard’ compounds since the compounds with the similarity <0.3 to every training compound were only about 10% of the unseen compounds (*n* = 28). Figure 3B and C shows the violin plots representing the distribution of *P*-values of true MoA pathways across 283 unseen compounds and 28 unseen-hard compounds respectively. According to Figure 3B and C, MoAble outperformed TS-978 and TS-12328 and showed performance comparable to TS-connectivity. We found MoAble was able to accurately predict the MoAs of even for the unseen compounds without using actual signatures.

We further investigated the effectiveness of MoAble on anticancer compounds as signaling pathways are usually misregulated in various cancers. Compounds with anatomical therapeutic chemical code (ATC) of ‘L01’ were selected as anticancer compounds. Eighty-eight out of 940 evaluation compounds were selected. Figure 3D shows the violin plots for the *P*-values of true MoA pathways across the anticancer compounds. We observed that a median *P*-value of 0 was achieved for both MoAble and TS-connectivity and a median *P*-value of 0.439 and 0.227 was achieved for TS-978 and TS-12328, respectively. These results highlight a distinctive improvement of connectivity-based methods (MoAble and TS-connectivity) compared to compound signature-based methods (TS-978 and TS-12328).

We validated MoAble for an additional 2674 compounds. We obtained the compounds from KEGG drug database. These compounds have MoA labels but without signatures in the L1000 dataset. Thus, only MoAble was able to predict MoAs for these compounds. Figure 3D illustrates the *P*-values of true MoA pathways for 2674 compounds (left) and for 94 anticancer compounds (right). Median *P*-value of 0.03 and 0 were achieved for 2674 compounds and anticancer compounds respectively. We highlight that MoAble consistently achieved low *P*-values for unseen compounds and showed improved prediction performance on anticancer compounds. Table 2 provides the median *P*-values of MoAble and baseline methods for each evaluation compound set. 

**Table 2.** Median *P*-value of the MoAble and baseline models across compounds sets

	MoAble	TS-connectivity	TS-978	TS-12328
Evaluation (#:940)	0.03	**0.01**	0.381	0.113
Unseen (#:283)	0.03	**0.01**	0.375	0.113
Unseen-hard (#:28)	0.017	**0.01**	0.406	0.106
Anticancer (#:88)	**0.0**	**0.0**	0.439	0.227
Novel (#:2674)	**0.03**	—	—	—
Novel anticancer (#:94)	**0**	—	—	—

Note: The lowest P-value for each evaluation compound set is highlighted in bold.

We further validated MoAble with expanded MoA labels. Following a similar approach as Ren *et al.* (2020) and Nguyen *et al.* (2019), we expanded MoA labels by including pathways that contain target proteins of compounds. Target proteins of compounds were downloaded from clue.io. After expanding MoA labels, we obtained a total of 1153 compounds for evaluation. We computed area under the receiver operating characteristic curve (AUROC) for each compound. AUROC is computed by calculating the true positive rate (TPR) against the false positive rate (FPR) with various FDR thresholds.

**Fig. 4.**
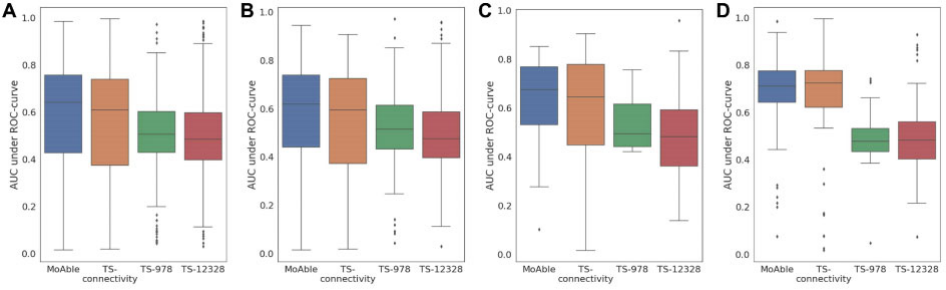
Area under the receiver operating characteristic (AUROC) of expanded MoA labels. The *x* axis represents each MoA prediction methods. (**A**) AUROC across 1153 evaluation compounds. (**B**) AUROC across 343 unseen compounds. (**C**) AUROC across 31 unseen-hard compounds. (**D**) AUROC across 55 anticancer compounds

Figure 4A illustrates box plots for the AUROC of 1153 compounds. MoAble (median AUROC = 0.642) achieved the highest AUROC scores, followed by TS-connectivity (median AUROC = 0.61), TS-978 (median AUROC = 0.507) and TS-12328 (median AUROC = 0.485). Also, MoAble achieved lower standard deviation than TS-connectivity (MoAble SD = 0.203, TS-connectivity SD = 0.213).

When we evaluated using only 343 unseen compounds, similar results were observed (Fig. 4B). MoAble (median AUROC = 0.62) achieved highest median AUROC, followed by TS-connectivity (median AUROC = 0.597), TS-978 (median AUROC = 0.516) and TS-12328 (median AUROC = 0.476). Moreover, MoAble (median AUROC = 0.677) achieved highest AUROC scores, followed by TS-connectivity (median AUROC = 0.646), TS-978 (median AUROC = 0.484) and TS-12328 (median AUROC = 0.496) on the 31 unseen-hard compounds (Fig. 4C). For 55 anticancer compounds out of 1153 compounds, MoAble significantly outperformed TS-978 and TS-12328. Moreover, MoAble was able to achieve similar accuracy with TS-connectivity, despite not using actual signatures. Table 3 provides the median AUROC scores of MoAble and baseline methods for each evaluation compound set. 

**Table 3.** Median AUROC of the MoAble and baseline models

	MoAble	TS-connectivity	TS-978	TS-12328
Evaluation (#1153)	**0.642**	0.610	0.507	0.485
Unseen (#343)	**0.620**	0.597	0.516	0.476
Unseen-hard (#31)	**0.677**	0.646	0.484	0.496
Anticancer (#55)	0.712	**0.725**	0.479	0.484

Note: The highest AUROC score for each evaluation compound set is highlighted in bold.

#### 3.2.3 Case study: identifying MoAs of SOS1-KRAS inhibitor

We conduct a case study with a drug candidate named BI-3406. It is designed for pan-KRAS inhibitor (Hofmann *et al.*, 2021). BI-3406 binds to SOS1, which is an upstream molecule of KRAS and inhibits KRAS activity by blocking the interaction between SOS1 and KRAS. Combined with MEK inhibitor (trametinib), BI-3406 showed effectiveness in KRAS-driven cancers (Hofmann *et al.*, 2021). The study provided compound signatures of BI-3406 (cell line: MIA-PaCa-2, timepoint: 4, 10 and 24 h, GEO accession number: GSE128385). Thus, we were able to compare MoA results obtained from MoAble with the ones obtained from actual signatures of BI-3406.

We examined whether MoAble was able to identify the RAS signaling pathway as a significant pathway. As shown in Table 4, MoAble successfully identified RAS signaling as a significant pathway at FDR < 0.1 (NES: 1.508, FDR: 0.0534). However, when we performed pathway enrichment analysis directly with the actual signature of BI-3406 (referred to as TS), it failed to identify RAS signaling as a significant pathway (NES: 1.208, *P*-value, FDR: 5309). In addition, MoAble was able to identify MAPK pathway, ERBB pathway (a downstream and an upstream pathway of KRAS, respectively) as significant pathways whereas prediction with TS failed to reveal these pathways. The case study shows MoAble accurately identified BI-3406 related MoAs whereas it fails when performing pathway enrichment analysis directly with actual signatures of BI-3406.

**Table 4.** MoA results of BI-3406 using MoAble and actual compound signature

	MoAble		TS	
Pathway	NES	FDR	NES	FDR
Ras signaling pathway	1.508	**0.0534**	1.208	0.5309
MAPK signaling pathway	1.889	**0.0035**	−1.546	0.1108
ErbB signaling pathway	2.334	**0.00012**	1.062	0.7038

Note: The lowest FDR for each pathway is highlighted in bold.

## 4 Discussion

CMap and LINCS-L1000 studies provide huge opportunities to reveal the MoAs of compounds. The large-scale signatures from CMap and LINCS-L1000 studies are useful resources to identify MoAs; the signatures consider the impact of compounds on transcriptomic phenotype. However, for the compounds whose signatures do not exist in CMap, LINCS-L1000 or other resources such as GEO repository, researchers are required to measure the signature experimentally, which is expensive and time-consuming. With the development of virtual screening, it can be utilized to screen more than 10 billion compounds in the early stage of drug discovery. However, it is almost impossible to experimentally measure the transcriptomic signatures of these compounds to reveal the MoA.

In this study, we present MoAble, a novel framework to identify MoAs of compounds without requiring actual signatures while taking into account the effect of compounds in transcriptional response. We used a coembedding model to jointly encode compound structures and signatures into the same latent embedding space. This allows the use of compound structures to generate embedding vectors representing the signature induced by the compound. To predict MoAs of a compound, GP signatures were mapped into the coembedding space. We computed the connectivity scores between compound structure embedding and GP signature embeddings. For a given compound, genes whose GP embeddings are highly connected to the compound structure embedding are considered to be potential targets of the compounds. We performed pathway enrichment analysis based on the connected GP genes to identify MoAs of compounds.

We first confirmed that the embedding vectors of compounds are predictive features for MoA prediction. We observed that the association between compounds and GP signatures was retained in the coembedding space. UMAP visualization showed that compounds with similar MoAs can be clustered in the coembedding space. To quantitatively evaluate the performance of MoA prediction, we compared MoAble to the three baseline methods that use actual signatures. We demonstrated that MoAble can predict MoAs with the same accuracy as that of using the actual signatures. Similar results were observed when we expanded MoA labels and used AUROC as the evaluation metric. We confirmed that predicting MoAs with the connected GP signature is more effective than using the compound signature directly. We also observed that connectivity-based methods are specifically more effective in anticancer compounds, than compound signature-based methods. Further studies are needed to establish the generalizability of connectivity-based MoA prediction for other diseases.

Future research can improve MoAble by utilizing more GP signatures. Increasing GP signatures can yield more robust pathway enrichment analysis by increasing the number of matched genes with pathway gene sets. In addition, MoAble can be improved by identifying MoAs a in cell-specific manner. This can be done by training the coembedding model to learn compound structure embeddings in a cell-type specific way. We expect MoAble to be useful in generating mechanistic hypotheses for novel compounds whose MoAs are unknown.

## Supplementary Material

btab275_Supplementary_Data
