# Supplementary material for: Predicting mechanism of action of novel compounds using compound structure and transcriptomic signature coembedding
Source: Bioinformatics. 2021 Jul 12;37(Suppl 1):i376–82. doi: 10.1093/bioinformatics/btab275 (PMC8275331; doi:10.1093/bioinformatics/btab275)
Supplement: btab275_Supplementary_Data [file btab275_supplementary_data.zip › MoAble_supplementary_data.docx]

**Predicting mechanism of action of novel compounds using compound structure and transcriptomic signature co-embedding**

**Supplementary Data**

Gwanghoon Jang^1^, Sungjoon Park^1,∗^, Sanghoon Lee^1^, Sunkyu Kim^1^, Sejeong Park^1^ and Jaewoo Kang^1,2,∗^

^1^Department of Computer Science and Engineering, Korea University, Seoul, Republic of Korea

^2^Interdisciplinary Graduate Program in Bioinformatics, Korea University, Seoul, Republic of Korea.

^∗^To whom correspondence should be addressed.


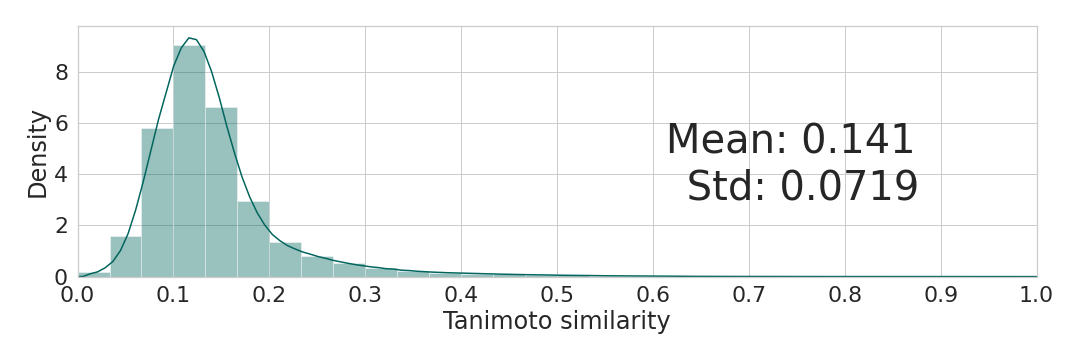


Figure S1. Distribution of Tanimoto similarity between training compounds and unseen compounds.
